# *bla*_NDM-5_ carried by a hypervirulent *Klebsiella pneumoniae* with sequence type 29

**DOI:** 10.1186/s13756-019-0596-1

**Published:** 2019-08-19

**Authors:** Yi Yuan, Ying Li, Guangxi Wang, Chengwen Li, Yung-Fu Chang, Wenbi Chen, Siji Nian, Yingyu Mao, Jinping Zhang, Fangcai Zhong, Luhua Zhang

**Affiliations:** 1 Department of Laboratory Medicine, The First People’s Hospital of Neijiang, Neijiang, Sichuan China; 2 Department of Immunology, School of Basic Medical Sciences, Southwest Medical University, Luzhou, Sichuan China; 3 Department of Pathogenic Biology, School of Basic Medical Sciences, Southwest Medical University, No.1 Section 1, Xiang Lin Road, Longmatan District, Luzhou, 646000 Sichuan China; 4 Department of Population Medicine and Diagnostic Sciences, College of Veterinary Medicine, Cornell University, Ithaca, NY USA

**Keywords:** Carbapenem resistance, *bla*_NDM-5_, Hypervirulent, IncX3

## Abstract

**Background:**

A carbapenem-resistant hypermucoviscous *Klebsiella pneumoniae* isolate was recovered from human sputum.

**Methods:**

Whole genome sequencing of this isolate was carried out to reveal its clonal background, antimicrobial resistance determinants and virulence factors. Virulence assays were performed using wax moth larvae. The transfer of *bla*_NDM-5_ between bacterial strains was tested using conjugation. 59 genome assemblies of ST29 *K. pneumoniae* and 230 IncX3 plasmids regardless of the carriage of resistance gene were employed for phylogenetic analysis, respectively.

**Results:**

The strain carried a virulence plasmid pVir-SCNJ1 bearing the virulence gene *rmpA* and exhibited a high virulence in wax moth. This hypervirulent strain belongs to sequence type 29 and carries *bla*_NDM-5_, which is located on a conjugative plasmid, designated pNDM5-SCNJ1, belonging to type IncX3. pNDM5-SCNJ1 was fully sequenced and shows high similarity with pNDM_MGR194, except some deletion inside the IS*Aba125* region. Phylogenetic analysis of IncX3 plasmids revealed that although *bla*_NDM-5_ can be evolved from *bla*_NDM-1_ via point mutations within some IncX3 plasmids, most of *bla*_NDM-5_-carrying IncX3 plasmids probably have acquired *bla*_NDM-5_ in multiple events.

**Conclusions:**

In this study, we characterized a *bla*_NDM-5_-positive hypervirulent *K. pneumoniae* of sequence type 29 in China. Our results highlight the need for active surveillance on this lineage of carbapenem-resistant *K. pneumoniae*.

**Electronic supplementary material:**

The online version of this article (10.1186/s13756-019-0596-1) contains supplementary material, which is available to authorized users.

## Introduction

Hypervirulent *Klebsiella pneumoniae* (hvKP) is a worldwide concern due to its capacity to cause life-threatening, community-acquired infections in healthy individuals with high morbidity and mortality [1, 2]. hvKP strains are usually less resistant to most antimicrobials than classic *K. pneumoniae* [3], but the increasing emergence of carbapenemase-producing hypervirulent *K. pneumoniae* (CP-hvKP) compromises options of antimicrobial agents for infection control and drives a global crisis [2, 4]. These CP-hvKP strains are thought to be the result of acquiring plasmid-mediated resistance and virulence markers, either by transferring of resistance plasmids into hvKP strains or virulence plasmids into carbapenem-resistant strains [5]. There have now been several reports of infections caused by carbapenemase-producing hypervirulent *K. pneumoniae* strains [2, 4, 6–9]. These CP-hvKP isolates mainly produce KPC (a group of serine-lactamases), followed by IMP (a group of metallo-β-lactamases), and they belong to the widely distributed sequence type (ST) 11 [2, 8] and several other STs, e.g., ST25, ST65 [6] and ST36 [4]. Here, we identified an ST29 CR-hvKP clinical strain with K54 serotype carrying *bla*_NDM-5_ gene and reported on its characterization.

## Materials and methods

### Bacterial identification and PCR analysis

The strain SCNJ1 was recovered from the sputum of a patient with an acute bronchiolitis in a hospital of Sichuan Province in November 2018. The initial species identification was performed using the Vitek-2 compact system (bioMérieux, Marcy-l’Étoile, France). A further species confirmation was performed by PCR amplifying of the 16S rRNA gene using the primer pair 27F/1492R [10]. PCR products were purified and then sequenced by Sanger sequencing. The resulting 16S rRNA gene sequences were compared with sequences in GenBank (NCBI) database using BLAST software. The presence of the acquired carbapenemase genes *bla*_KPC_, *bla*_NDM_, *bla*_GES_, *bla*_IMP_, *bla*_OXA-48_, and *bla*_VIM_ in this isolate was screened via PCR using primers as previously described [11–14].

### Antimicrobial susceptibility tests

In vitro susceptibility tests of cefepime, piperacillin-tazobactam, ampicillin, ampicillin/sulbactam, cefotetan, ceftriaxone, cefazolin, nitrofurantoin, ceftazidime, tobramycin, ciprofloxacin, trimethoprim/sulfamethoxazole, aztreonam, amikacin, gentamicin and levofloxacin were performed using Vitek-2 system. The minimum inhibitory concentrations (MICs) of imipenem, meropenem and colistin against the isolate were determined using the microdilution broth method following recommendations of the Clinical Laboratory Standards Institute (CLSI) [15]. Breakpoints of colistin was defined by the European Committee on Antimicrobial Susceptibility Testing (EUCAST) (http://www.eucast.org/), otherwise, we applied those defined by the CLSI.

### String test

String test was performed by stretching a mucoviscous string from the colony using a standard bacteriologic loop as described previously [16]. Strains that formed viscous strings *>* 5 mm in length were defined as hypermucoviscous.

### Conjugation

Conjugation experiments were carried out using broth-based methods with the azide-resistant *Escherichia coli* strain J53 as the recipient and transconjugants were selected using 2 μg/ml meropenem plus 150 μg/ml sodium azide. The presence of *bla*_NDM-5_ in transconjugants was confirmed by PCR and sequencing.

### Virulence assay

The virulence potential of the SCNJ1 strain was assessed using wax moth (*Galleria mellonella*) larvae weighing 250 to 350 mg (Tianjin Huiyude Biotech Company, Tianjin, China) with method described previously [17]. Overnight cultures of *K. pneumoniae* strains were adjusted with phosphate-buffered saline (PBS) to concentrations of 1 × 10^4^ CFU/ml, 1 × 10^5^ CFU/ml, 1 × 10^6^ CFU/ml, 1 × 10^7^ CFU/ml after being washed with PBS. 10 μl of inoculum was injected into the hemocoel of sixteen larvae using a 25-μl Hamilton syringe via the last left proleg. The larvae were then incubated at 37 °C and the number of live larvae was counted at 12 h intervals for 3 days. Two *bla*_KPC-2_-carrying carbapenem-resistant *K. pneumoniae* clinical isolates of ST11:K47, KPNJ2 and KPLZ1050, without *rmpA* and *rmpA2*, were used as the control.

### Genome sequencing and analysis

The strain was subjected to whole genomic sequencing using an Illumina HiSeq 2000 system with the 150-bp paired-end approach and 150 × coverage. Reads were trimmed using Trimmomatic [18]. Draft genome was then assembled using the SPAdes program [19]. Annotation was carried out using Prokka [20]. Sequence type and capsular type of this strain were determined using the assembled contigs to query the Multi-Locus Sequence Typing (MLST) v 2.0 (https://cge.cbs.dtu.dk/services/MLST/) and *wzc* genotyping system as previously described [21], respectively. In addition, the *wzi* genotyping system [22] and KLeborate (https://github.com/katholt/Kleborate/) were employed to confirm the sequence type and capsular type. Clonal complexes (CCs) were determined by using eBURST v3 based on *K. pneumoniae* MLST data (https://eburst.mlst.net). Virulence genes were identified using the **V**irulence **F**actors **D**ata**b**ase (VFDB) available at http://www.mgc.ac.cn/VFs/main.htm. Antimicrobial resistance genes were identified using the ResFinder v3.1 software of the Center for Genomic Epidemiology (CGE, http://genomicepidemiology.org/).

Plasmids pVir-SCNJ1 and pNDM5-SCNJ1 were completely circularized with gaps between the contigs closed by PCR and respective amplicons sequenced using Sanger sequencing, respectively. Identification of plasmid incompatibility types were performed on complete sequences of plasmids via the online service PlasmidFinder v2.0 at CGE (https://cge.cbs.dtu.dk/services/PlasmidFinder/). The annotations of the plasmid sequences were conducted using the RAST tools and edited manually [23]. Sequence alignment of *bla*_NDM-5_-carrying plasmids was performed using BLAST and visualized with Easyfig v 2.2.3 [24]. Alignments with highly homologous complete plasmid sequences of pVir-SCNJ1 available in NCBI were performed by using the BRIG tool [25]. The circular map of pNDM5-SCNJ1 was also generated using BRIG [25].

### Phylogenetic analysis

All assembled *K. pneumoniae* genomes (*n* = 6823; accessed by March 1, 2019) were retrieved from GenBank. MLST typing was performed using the script (https://github.com/tseemann/mlst). A total of 59 assemblies of ST29 *K. pneumoniae* were included and aligned with that of strain SCNJ1 using CSI Phylogeny 1.4 (https://cge.cbs.dtu.dk/services/CSIPhylogeny/) (Additional file 1 :Table S2). Gubbins (version 2.3.4) was used to remove single nucleotide polymorphisms (SNPs) on recombination sites [26]. The filtered SNPs were then used as input for inferring a phylogenetic tree using RAxML with the GTRGAMMA model and 1000 bootstraps [27]. ABRicate (https://github.com/tseemann/abricate) was used to identify antimicrobial resistance genes in these genomes and the capsular typing of *K. pneumoniae* was performed with the *wzc* genotyping system.

The sequence of all available IncX3 plasmids regardless of the carriage of resistance gene (*n* = 230; accessed by March 24, 2019) were retrieved from the GenBank (Additional file 1 :Table S4). Orthogroups were identified using OrthoFinder [28] and used for multiple sequence alignments (MSA) with MAFFT [29]. The species tree was inferred from the concatenated MSA using FastTree [30]. The STRIDE algorithm (Specie Tree Root Inference from Duplication Events) was used to root the species tree in OrthoFinder.

## Results and discussion

Antimicrobial susceptibility test showed that the *K. pneumoniae* SCNJ1 strain was resistant to imipenem (MIC, > 256 μg/ml) and meropenem (MIC, > 256 μg/ml) cefepime (MIC, 16 μg/ml), piperacillin-tazobactam (MIC, ≥ 128 μg/ml), ampicillin (MIC, ≥ 32 μg/ml), ampicillin/sulbactam (MIC, ≥ 32 μg/ml), cefotetan (MIC, ≥ 64 μg/ml), ceftriaxone (MIC, ≥ 64 μg/ml), cefazolin (MIC, ≥ 64 μg/ml), nitrofurantoin (MIC,128 μg/ml) and ceftazidime (MIC, ≥ 64 μg/ml), but was susceptible to colistin (MIC, 2 μg/ml), tobramycin (MIC, ≤ 1 μg/ml), ciprofloxacin (MIC, ≤ 0.25 μg/ml), trimethoprim/sulfamethoxazole (MIC, ≤ 20 μg/ml), aztreonam (MIC, ≤ 1 μg/ml), amikacin (MIC, ≤2 μg/ml), gentamicin (MIC, ≤1 μg/ml) and levofloxacin (MIC, ≤ 0.25 μg/ml). Strain SCNJ1 showed hypermucoviscosity phenotype as evidenced by forming a viscous string about 35 mm, which is beyond the > 5 mm to define hypermucoviscous.

PCR and sequencing showed that *bla*_NDM-5_ was the only carbapenemase-encoding gene carried by the strain SCNJ1. NDM-5, a variant of NDM (New Delhi Metallo-β-lactamase), was first identified in an *E. coli* ST648 isolate (EC045) in the UK in 2011 from a patient with a recent hospitalization history in India [31]. Although *bla*_NDM-5_ has been widely found in *K. pneumoniae* strains since its first discovery (Additional file 1 :Table S1), *bla*_NDM-5_-carrying hypermucoviscous *K. pneumoniae* remains uncommon. We found one publication that described a *bla*_NDM-5_-positive *K. pneumoniae* isolate (K2/ST14) in China in 2015 [32], which was speculated to be hypermucoviscous on the basis of genome analysis. However, no experimental data was included.

Draft genome sequences of SCNJ1 was assembled into 29 contigs (28 were > 1000 bp in length), which comprises 5,474,953 bp, with a 57.29% GC content. SCNJ1 was assigned to capsular type *wzi*115-K54 and sequence type ST29 (*gapA-infB-mdh-pgi-phoE-rpoB-tonB* allele number 2–3–2-3-6-4-4). K54 is a hypervirulent member of *K. pneumoniae* [16] and has been described in several previous publications as linked to ST29 [33–37]. To date, *K. pneumoniae* strains with the ST29 group has a worldwide distribution and has been found carrying a variety of carbapenem genes, including *bla*_NDM-1_ [38–40], *bla*_KPC_ [41], *bla*_OXA-48_ [42] and *bla*_OXA-181_ [38], as well as several extended-spectrum β-lactamases (ESBLs) genes [43, 44] in various countries. However, the currently available evidence is insufficient to demonstrate whether ST29, a member of CC29, is an epidemic clone mediating the spread of specific and clinically relevant antibiotic resistance genes.

**Fig. 1:**
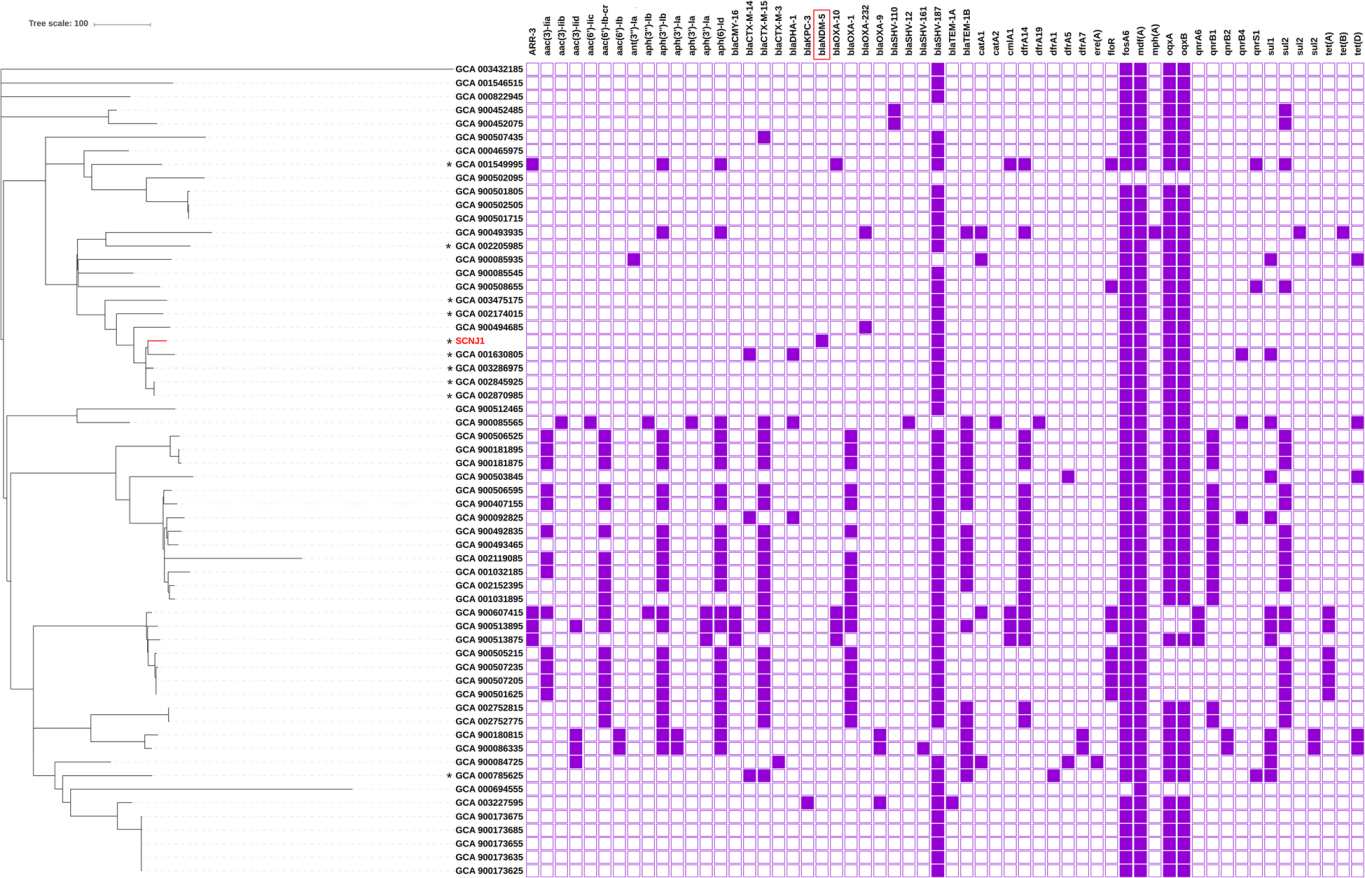
Phylogenetic tree and resistance gene profile of *K. pneumoniae* strain SCNJ1 with other 59 ST29 *K. pneumoniae* genomes available from GenBank. SCNJ1 is indicated in red. Isolates from China are marked with an asterisk and resistance gene *bla*_NDM-5_ is marked with a red rectangle

The gene *bla*_NDM-5_ is described for the first time in a strain of *K. pneumoniae* ST29 in our work, as demonstrated by the phylogenetic tree based on filtered SNPs of all available ST29 *K. pneumoniae* strains (Fig. 1). The phylogenetic analysis also showed that strain SCNJ1 was clustered with four isolates recovered in China and was closest to strain SCLZ15–011 (GCA_001630805, carrying no carbapenemase gene, recovered in 2016 in China) with 198 SNPs difference (Fig. 1). It should be noted that sporadic cases due to ST29 *K. pneumoniae* were frequently detected, mainly from liver abscess patients [37, 43, 45, 46], and multidrug-resistant ST29 hvKP strains have been reported in different locations of China [5, 33, 47]. This highlights the need to monitor the epidemiology of the ST29 clones of *K. pneumoniae* isolates in China.

**Fig. 2:**
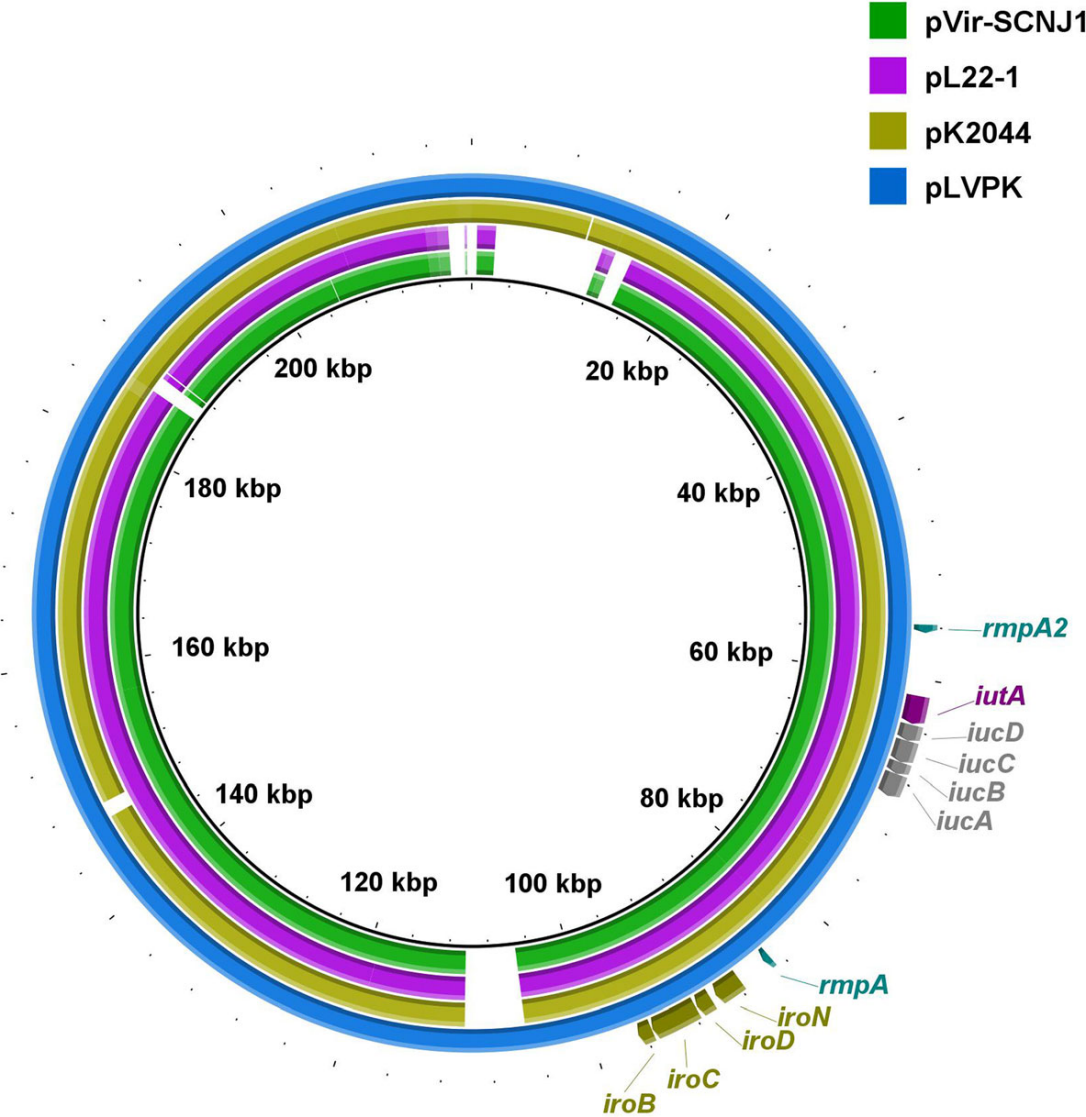
Alignment of pVir-SCNJ1 with 3 hypervirulence-encoding plasmids. The alignment was performed using BRIG and pLVPK was used as a reference. Accession numbers for the plasmids are NZ_CP031258 (pL22–1), NC_006625 (pK2044), NC_005249 (pLVPK). The locations of virulence genes *rmpA2*, *iutA*, *iucDCBA*, *rmpA* and *iroNDCB* are indicated

**Fig. 3:**
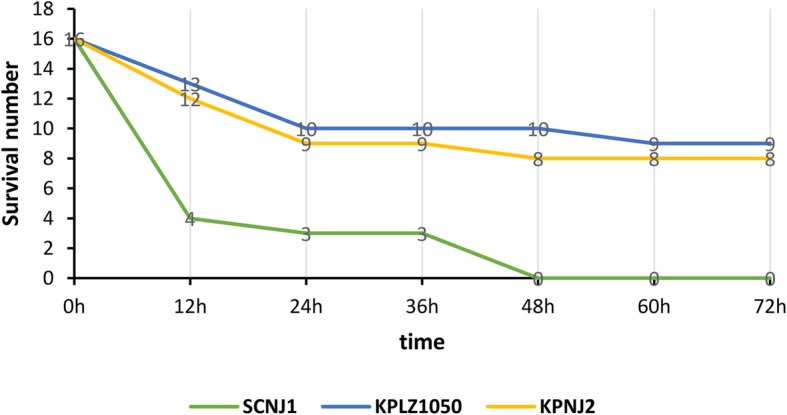
Virulence potential of *K. pneumoniae* strains in a *G. mellonella* infection model. The effect of 1 × 10^5^ CFU/ml of each isolate on survival of *G. mellonella* is shown. The results for other doses of each *K pneumoniae* strain are shown in Supplementary Table S3. KPLZ1050 and KPNJ2, two *bla*_KPC − 2_-carrying *K pneumoniae* clinical isolates of ST11 that did not harbour a virulence plasmid*,* were used as the control

We found that strain SCNJ1 harbored genes encoding regulators of the mucoid phenotype (*rmpA*), aerobactin (*iuc*ABCD and *iut*A), ent siderophore (*ent*ABCDEFS, *fep*ABCDG), salmochelin (*iro*BCDEN), yersiniabactin (*fyu*A, *irp*1, *irp*2 and *ybt*AEPQSTUX) and type 3 fimbriae (*mrkABCDFHIJ*) etc. These genes are frequently associated with hypervirulence phenotype of *K. pneumoniae* [16, 48]*.* The *rmpA*, *iutA, iucABCD* and *iroBCDN* genes were carried by a 211,807-bp plasmid, designated pVir-SCNJ1. The *rmpA2* (another regulator of mucoid phenotype) gene on the pVir-SCNJ1 plasmid was truncated, due to a frameshift mutation introducing an internal stop codon. pVir-SCNJ1 was an IncHI1/IncFIB-type plasmid and was 99.71% identical to the known virulence plasmid pLVPK (219,385 bp, GenBank accession no. NC_005249) at 93% coverage [49] (Fig. 2). It was notable that pVir-SCNJ1 was highly similar (99% coverage and 99.99% identity, Fig. 2) to the recently-identified plasmid pL22–1 (212, 635 bp, GenBank accession no. NZ_CP031258) that recovered from a *Klebsiella quasipneumoniae* strain L22, which suggests that the pLVPK-like plasmid has the potential to mediate inter- and intra-species transfer of virulence genes [50]. Our virulence assays showed that survival of *G. mellonella* was 0% with strains SCNJ1, while survival was 56.2 and 50.0% with the control strains KPLZ1050 and KPNJ2, at an inoculum of 1 × 10^5^ cfu/mL at 72 h after infection, respectively (Fig. 3, Additional file 1 :Table S3). These findings suggest that strain SCNJ1 was hypervirulent.

Conjugation assays showed that strain SCNJ1 transferred a plasmid carrying *bla*_NDM-5_ to *E. coli* J53 at a frequency of 10^− 6^ (transconjugant/recipient) by mating, suggesting that *bla*_NDM-5_ was carried on a self-transmissible plasmid, which was assigned pNDM5-SCNJ1. In addition to the *bla*_NDM-5_, strain SCNJ1 had a few chromosomal resistance genes, including the ESBL gene *bla*_SHV-187_, fluoroquinolone-resistance genes *oqxA* and *oqxB,* and fosfomycin-resistance gene *fosA*.

**Fig. 4:**
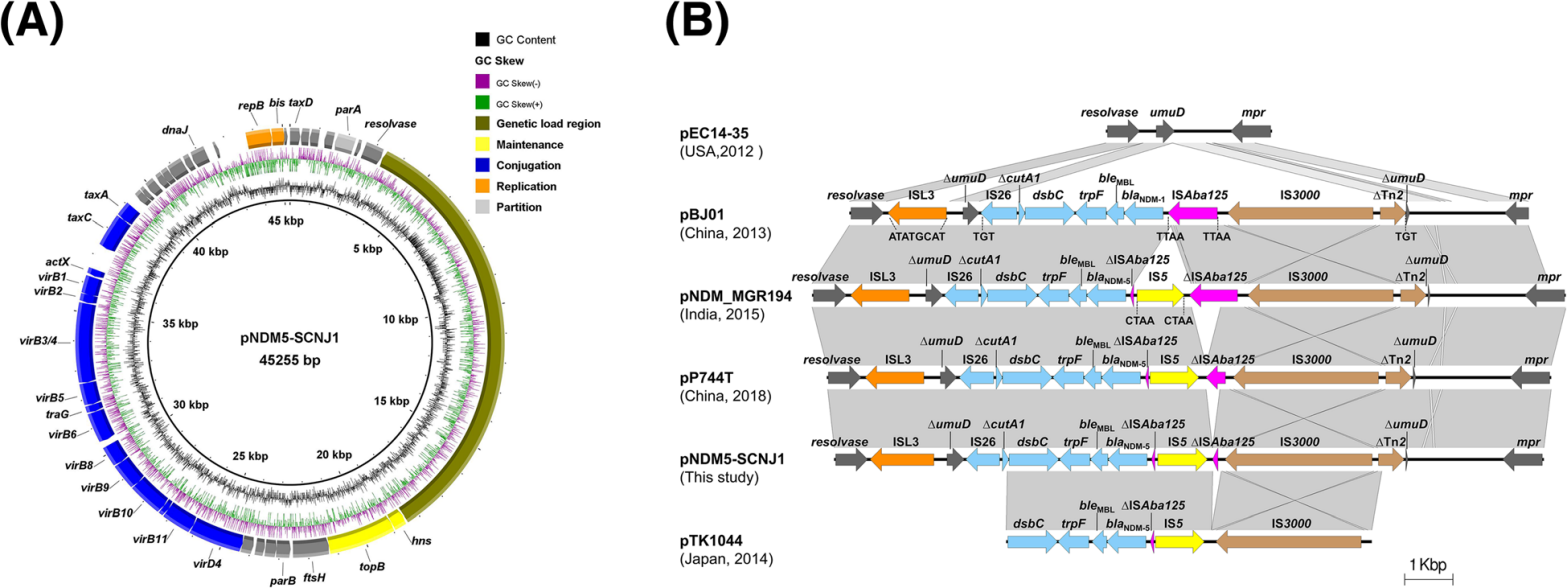
Plasmid analysis of pNDM5-SCNJ1. (**a**) Genetic structure of IncX3 plasmid pNDM5-SCNJ1. This map was used to illustrate the backbone and the location of the genetic load region of pNDM5-SCNJ1. Genes are denoted by arrowheads and colored based on gene function classification. (**b**) Comparative analysis of the genetic load region of pNDM5-SCNJ1. Genes and insertion sequences are indicated by arrows. Light gray shades denote shared regions with a high degree of homology. The accession numbers were: pEC14_35 (JN935899), pBJ01(JX296013), pNDM_MGR194 (KF220657), pP744T (MF547511), pTK1044 (LC000627). The alignment is a pairwise BLASTn alignment performed using Easyfig [24]

pNDM5-SCNJ1 was a 45,255-bp IncX3 plasmid, with an average GC content of 46.83% and had no other known antimicrobial resistance genes except *bla*_NDM-5_. pNDM5-SCNJ1 consists of a 30-kb backbone comprising several sets of genes (*pir* and *bis* encoding replication initiation protein, *parA* for plasmid partitioning, *hns* and *topB* for maintenance and a gene cluster responsible for conjugation) and a genetic load region with high GC content between the resolvase and the *hns* gene, which are typical of IncX3 plasmids (Fig.4a). BLASTn revealed that the sequence of pNDM5-SCNJ1 was highly similar (100% coverage and 99.99% identity) to the plasmid pNDM_MGR194 (GenBank accession no. KF220657) recovered from a *K. pneumoniae* isolate in India, as well as a number of previously described IncX3 plasmids carrying *bla*_NDM-5_ in China.

In the genetic load region of pNDM5-SCNJ1(Fig. 4b), the *umuD* gene was split into two fragments at the nucleotide position 336 bp by the *bla*_NDM-5_-containing structure (IS*26*-Δ*ctuA1*-*tat*-*trpF*-*ble*_MBL_-*bla*_NDM-5_-ΔIS*Aba125*-IS*5*-ΔIS*Aba125*-IS*3000*-ΔTn2), resulting in a pair of 3-bp direct repeats (TGT). In such a genetic context, an IncX3 plasmid pEC14_35 (GenBank accession no. JN935899) without any antibiotic-resistance gene, which was isolated from a patient in the USA in 1989, was likely to be the ancestral vector. It is also likely that pNDM5-SCNJ1 has diverged recently from *bla*_NDM-1_-positive plasmids pBJ01 (GenBank accession no. JX296013) by sequential mutations (Fig. 4b). In the subsequent genetic variant, the IS*Aba125* was truncated by the insertion of IS*5* element (at 166 bp upstream *bla*_NDM-5_ start codon) and a 4-bp flanking direct repeats (CTAA) was identified. Comparisons of the genetic contexts of *bla*_NDM-5_ in pNDM_MGR194, pP744T, pTK1044 and pNDM5-SCNJ1 showed that the remnant of IS*Aba125* (73 bp of 1087 bp) upstream of *bla*_NDM-5_ was conserved, but the length of the remnant of IS*Aba125* between IS*3000* and IS*5* differed (pNDM_MGR19: 1002 bp, pP744T: 404 bp, pNDM-SCNJ1: 112 bp, pTK1044: 0 bp), suggesting that IS*5* has inserted into IS*Aba125* at the same position in these plasmids and that gene deletions caused by homologous recombination plays a possible role in the formation of diversified ΔIS*Aba125* region.

IncX3 plasmids are narrow-host-range vectors of the *Enterobacteriaceae* [51, 52]. Searches on IncX3 plasmids in NCBI showed that they were recovered from various species of *Enterobacteriaceae* (Additional file 1 :Table S4), including *E. coli*, *K. pneumoniae*, *Citrobacter freundii*, *Enterobacter cloacae*, *Klebsiella oxytoca*, *Enterobacter hormaechei*, *Salmonella enterica, Kluyvera intermedia*, *Morganella morganii*, *Raoultella planticola* and *Raoultella ornithinolytica* from different countries, suggesting a wide distribution of IncX3 plasmids. Three kinds of carbapenemase genes were found to be carried by IncX3 plasmids, including *bla*_NDM_, *bla*_OXA-181_ and *bla*_KPC_. The carriage rate of *bla*_NDM_ was significantly higher (*n* = 150, 64.94%) than those of *bla*_KPC_ (*n* = 18, 7.79%) and *bla*_OXA-181_ (*n* = 17, 7.35%) (Additional file 1 :Table S4). Of note, IncX3 plasmids were found to carry many different *bla*_NDM_ alleles, including NDM-1, 4, 5, 6, 7, 13, 19, 20, 21, which were mainly recovered from China, confirming that IncX3 plasmids function as a common vehicle in facilitating the rapid dissemination of NDM-type MBLs among Enterobacteriaceae in China.

**Fig. 5:**
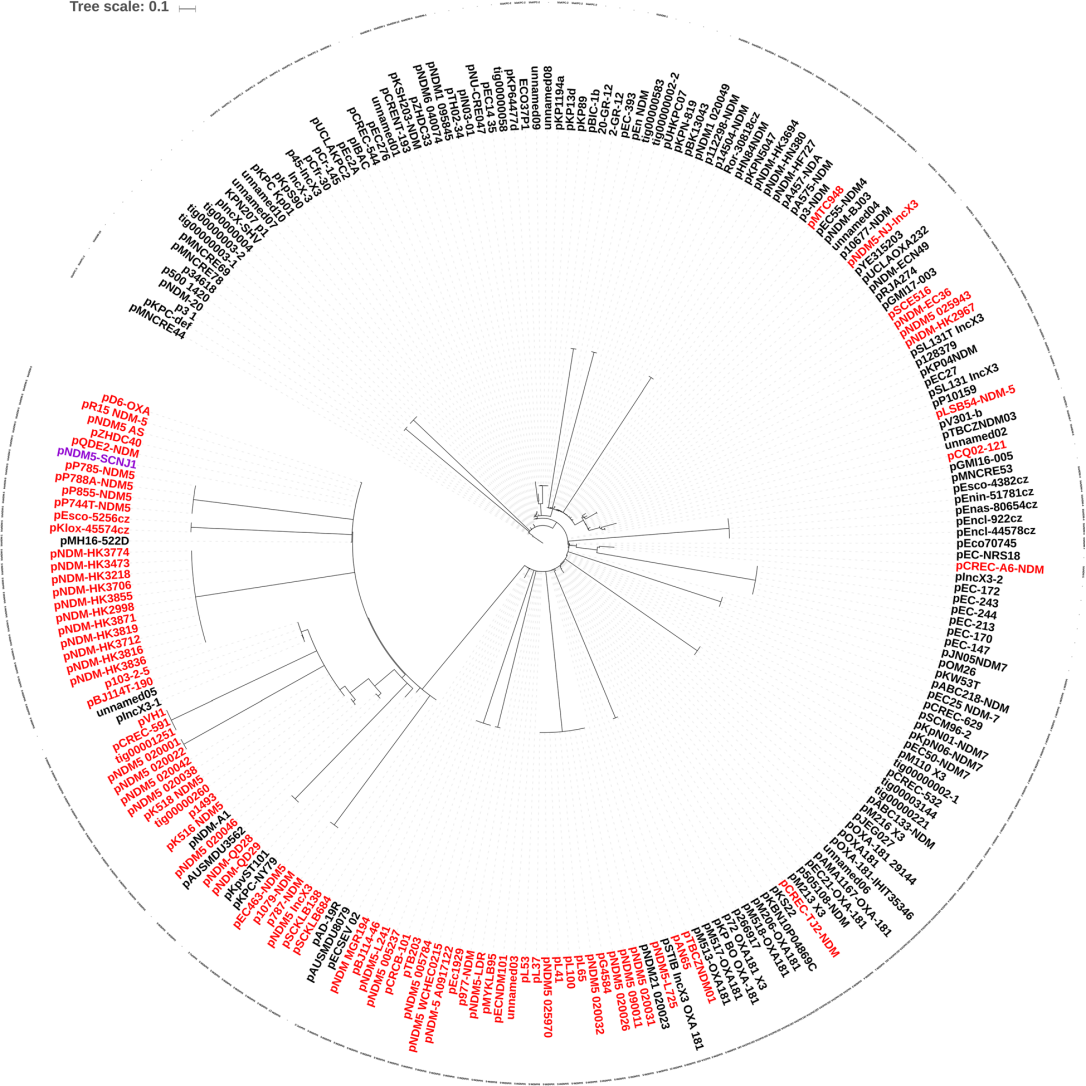
Phylogenetic analysis of IncX3 plasmids. Detailed information of the plasmids is shown in Supplementary Table 4. Those carrying *bla*_NDM-5_ are indicated in red, while pNDM5-SCNJ1 is shown in purple. The species tree was inferred from the concatenated multiple sequence alignments using FastTree

Phylogenetic analysis based on concatenated MSA of IncX3 plasmids revealed that pNDM5-SCNJ1 was closely related (100% coverage, 99.99% identity) to plasmid pQDE2-NDM (GenBank accession no. MH917280), which also carried *bla*_NDM-5_ that was recovered from a *K. pneumoniae* isolate in Shandong, China, in 2015 (Fig. 5). The phylogenetic tree also showed that most of *bla*_NDM-5_-carrying IncX3 plasmids are tightly clustered with each other and formed a relatively distinct clade, with only sporadic ones clustered into clades with *bla*_NDM-1_-carrying plasmids, suggesting that although *bla*_NDM-5_ is likely to evolve from *bla*_NDM-1_ via point mutations on some IncX3 plasmids, most of IncX3 plasmids probably have acquired *bla*_NDM-5_ in multiple events.

## Conclusion

In conclusion, our work identified an ST29 CP-hvKP carrying the carbapenemase gene *bla*_NDM-5_ and provided additional evidence of the rapid dissemination of *bla*_NDM-5_ by pNDM-MGR194-like plasmid among *Enterobacteriaceae* in China. The association of the epidemic IncX3 plasmid carrying *bla*_NDM-5_ with a hypervirulent *K. pneumoniae* lineage, ST29/K54 in this case, is quite worrisome and may pose a great threat to humans. More extensive surveillance and effective action to control its further dissemination are urgently required.

## Additional file

Additional file 1: **Table S1.** Background information on the *bla*_NDM-5_-positive *K. pneumoniae* isolates. **Table S2.** ST29 *K. pneumoniae* strains with genome sequences available in the GenBank. **Table S3.** Survival (number of larvae) of *G. mellonella* after infection by *K. pneumoniae* strain SCNJ1. **Table S4.** The names, host species, accession numbers, carbapenemase genes and locations of IncX3 plasmids. (DOCX 109 kb)

## Data Availability

Draft whole-genome sequences of the SCNJ1 strain has been deposited into GenBank under the accession no. SPSD00000000. The complete sequences of pVir-SCNJ1 and pNDM5-SCNJ1 have been deposited into GenBank under accession no. MK715436 and MK715437, respectively.
