# Supplementary material for: blaNDM-5 carried by a hypervirulent Klebsiella pneumoniae with sequence type 29
Source: Antimicrob Resist Infect Control. 2019 Aug 19;8:140. doi: 10.1186/s13756-019-0596-1 (PMC6701021; doi:10.1186/s13756-019-0596-1)
Supplement: Supplementary file 1 — Table S1. Background information on the blaNDM-5-positive K. pneumoniae isolates. Table S2. ST29 K. pneumoniae strains with genome sequences available in the GenBank. Table S3. Survival (number of larvae) of G. mellonella after infection by K. pneumoniae strain SCNJ1. Table S4. The names, host species, accession numbers, carbapenemase genes and locations of IncX3 plasmids. (DOCX 109 kb) [file 13756_2019_596_MOESM1_ESM.docx]

Table S1. Background information on the *bla*_NDM-5_-positive *K. pneumoniae* isolates

| Strain | Type of specimen /animal /infection | Sample origin | MLST type/ capsule serotype | Plasmid/ Plasmid type | Year | Country | Reference |
| --- | --- | --- | --- | --- | --- | --- | --- |
| MGR194 | NA | Blood | NA | IncX3 | 2011-2013 | India | [1] |
| KP1053 | Inpatient | Urine | ST231 | NA | 2012 | Singapore | [2] |
| CPO-2014-0020 | NA | Urine | ST16/k51 | IncX3 | 2014 | Denmark | [3] |
| CPO-2014-0029 | NA | Abscess, aspirate | ST16/k51 | IncX3 | 2014 | Denmark | [3] |
| CPO-2014-0032 | NA | Urine | ST16/k51 | IncX3 | 2014 | Denmark | [3] |
| CPO-2014-0041 | NA | Urinary, catheter | ST16/k51 | IncX3 | 2014 | Denmark | [3] |
| CPO-2015-0003 | NA | Respiratory secretion | ST16/k51 | IncX3 | 2015 | Denmark | [3] |
| NL–15360 032831 | spinal cord injury | rectal swab | ST16 | NA | 2015 | Netherlands | [4] |
| CC1409-1 | aspiration pneumonia | bronchoalveolar lavage ﬂuid | ST147 | NA | 2014 | South Korea | [5] |
| CC1410-1 | traumatic intracranial hemorrhage | tracheal aspirate | ST147 | NA | 2014 | South Korea | [5] |
| KPE127 | pneumonia | endotracheal tube swab | ST45 | NA | 2014 | Egypt | [6] |
| KPB140 | septicemia | blood | ST45 | NA | 2014 | Egypt | [6] |
| SZ03 | Inpatient | Surgery wound | ST25 | IncX3 | 2016 | China | [7, 8] |
| SZ04 | Inpatient | Peritoneal fluid | ST25 | IncX3 | 2016 | China | [7, 8] |
| NUHL24835 | Inpatient | urine | ST14/K2 | IncX3 | 2015 | China | [9] |
| NA | pyelonephritis | urine | ST340 | NA | NA | Spain | [10] |
| XG-K3 | dairy cows | faeces | ST1661 | IncX3 | 2015 | China | [11] |
| XG-K4 | dairy cows | faeces | ST1661 | IncX3 | 2015 | China | [11] |
| XG-K9 | dairy cows | faeces | ST1661 | IncX3 | 2015 | China | [11] |
| TQ-K1 | dairy cows | faeces | ST2108 | IncX3 | 2015 | China | [11] |
| TQ-K2 | dairy cows | faeces | ST2108 | IncX3 | 2015 | China | [11] |
| TQ-K3 | dairy cows | faeces | ST17 | IncX3 | 2015 | China | [11] |
| TQ-K4 | dairy cows | faeces | ST2108 | IncX3 | 2015 | China | [11] |
| K11424 | dairy cows | raw milk | ST2108 | IncX3 | 2015 | China | [11] |
| K12016 | dairy cows | raw milk | ST530 | IncX3 | 2015 | China | [11] |
| YZ-K1 | dairy cows | faeces | ST2293 | IncX3 | 2015 | China | [11] |
| NA | Inpatient | faecal, rectal screening swab, catheter urine | ST2266 | NA | 2015 | New Zealand | [12] |
| NA | Inpatient | NA | ST1 | NA | 2012-2015 | China | [13] |
| CRKP-1215 | NA | Bronchial washing | ST147 | IncFII | 2010 - 2015 | South Korea | [14] |
| CRKP-2297 | NA | Bronchial washing | ST147 | IncFII | 2010 - 2015 | South Korea | [14] |

NA: not available;

Table S2. ST29 *K. pneumoniae* strains with genome sequences available in the GenBank.

| Assembly no. | Accession no. | Recovery date | Location | Capsular type |
| --- | --- | --- | --- | --- |
| GCA_000465975 | NZ_CP012883 | 2015 | Singapore | K54 |
| GCA_000694555 | NZ_KK737551 | 2014 | USA | K30 |
| GCA_000785625 | NZ_KN360959 | 2014 | China | K30 |
| GCA_000822945 | NZ_CCJB01000001 | 2014 | Italy | NA |
| GCA_001031895 | NZ_KQ088519 | 2015 | USA | K19 |
| GCA_001032185 | NZ_KQ088661 | 2015 | USA | K19 |
| GCA_001546515 | NZ_KQ956885 | 2016 | USA | K30 |
| GCA_001549995 | NZ_LROW01000001 | 2015 | China | K54 |
| GCA_001630805 | NZ_LWLM01000001 | 2016 | China | K54 |
| GCA_002119085 | NZ_NBYZ01000069 | 2017 | Brazil | K19 |
| GCA_002152395 | NZ_NGST01000001 | 2017 | USA | K19 |
| GCA_002174015 | NZ_MOSR01000001 | 2017 | China | K54 |
| GCA_002205985 | NZ_MOMG01000001 | 2017 | China | K54 |
| GCA_002752775 | NZ_CP024489 | 2017 | Australia | K30 |
| GCA_002752815 | NZ_CP024482 | 2017 | Australia | K30 |
| GCA_002845925 | NZ_CP025091 | 2017 | China | NA |
| GCA_002870985 | CP025629 | 2018 | China | NA |
| GCA_003227595 | PQUL01000100 | 2018 | Italy | K30 |
| GCA_003286975 | CP030172 | 2018 | China | K54 |
| GCA_003432185 | CP031808 | 2018 | Australia | K20 |
| GCA_003475175 | QROX01000001 | 2018 | China | K54 |
| GCA_900084725 | NZ_FLCZ01000001 | 2016 | United Kingdom | K30 |
| GCA_900085545 | NZ_FLGD01000001 | 2016 | United Kingdom | K54 |
| GCA_900085565 | NZ_FLGH01000001 | 2016 | United Kingdom | K30 |
| GCA_900085935 | NZ_FLHR01000001 | 2016 | United Kingdom | K54 |
| GCA_900086335 | NZ_FLJF01000001 | 2016 | United Kingdom | K30 |
| GCA_900092825 | NZ_FLVS01000034 | 2016 | United Kingdom | K19 |
| GCA_900173625 | NZ_FWMK01000058 | 2017 | United Kingdom | K30 |
| GCA_900173635 | NZ_FWMN01000059 | 2017 | United Kingdom | K30 |
| GCA_900173655 | NZ_FWMS01000059 | 2017 | United Kingdom | K30 |
| GCA_900173675 | NZ_FWMQ01000056 | 2017 | United Kingdom | K30 |
| GCA_900173685 | NZ_FWMJ01000054 | 2017 | United Kingdom | K30 |
| GCA_900180815 | NZ_FXMT01000053 | 2017 | United Kingdom | K30 |
| GCA_900181875 | NZ_FXRK01000052 | 2017 | United Kingdom | K19 |
| GCA_900181895 | NZ_FXRH01000047 | 2017 | United Kingdom | K19 |
| GCA_900407155 | OVDU01000107 | 2018 | Tanzania | K19 |
| GCA_900452075 | UGMA01000005 | 2018 | United Kingdom | K8 |
| GCA_900452485 | UGNE01000002 | 2018 | United Kingdom | K8 |
| GCA_900492835 | UFBG01000046 | 2018 | United Kingdom | K19 |
| GCA_900493465 | UFDL01000037 | 2018 | United Kingdom | K19 |
| GCA_900493935 | UFER01000058 | 2018 | United Kingdom | K54 |
| GCA_900494685 | UFHT01000045 | 2018 | United Kingdom | K54 |
| GCA_900501625 | UIVA01000060 | 2018 | United Kingdom | K30 |
| GCA_900501715 | UIVO01000076 | 2018 | United Kingdom | K54 |
| GCA_900501805 | UIVU01000079 | 2018 | United Kingdom | K54 |
| GCA_900502095 | UIWW01000077 | 2018 | United Kingdom | K54 |
| GCA_900502505 | UIYN01000059 | 2018 | United Kingdom | K54 |
| GCA_900503845 | UJDV01000380 | 2018 | United Kingdom | K19 |
| GCA_900505215 | UJIX01000168 | 2018 | United Kingdom | K30 |
| GCA_900506525 | UJNY01000071 | 2018 | United Kingdom | K19 |
| GCA_900506595 | UJOF01000055 | 2018 | United Kingdom | K19 |
| GCA_900507205 | UJQS01000074 | 2018 | United Kingdom | K30 |
| GCA_900507235 | UJQQ01000244 | 2018 | United Kingdom | K30 |
| GCA_900507435 | UJRP01000435 | 2018 | United Kingdom | K10 |
| GCA_900508655 | UJWK01000233 | 2018 | United Kingdom | K54 |
| GCA_900512465 | UKKY01000065 | 2018 | United Kingdom | K30 |
| GCA_900513875 | UKQG01000072 | 2018 | United Kingdom | K30 |
| GCA_900513895 | UKRD01000070 | 2018 | United Kingdom | K30 |
| GCA_900607415 | UWVD01000006 | 2018 | Switzerland | K30 |

NA: not available;

Table S3. Survival (number of larvae) of *G. mellonella* after infection by *K. pneumoniae* strain SCNJ1.

| Strain | Inoculum (CFU/ml) | | | |
| --- | --- | --- | --- | --- |
|  | 1 × 10^4^ | 1 × 10^5^ | 1 × 10^6^ | 1 × 10^7^ |
| SCNJ1 |  |  |  |  |
| 12h | 5 | 4 | 3 | 1 |
| 24h | 5 | 3 | 2 | 0 |
| 36h | 3 | 3 | 0 | 0 |
| 48h | 3 | 0 | 0 | 0 |
| 60h | 3 | 0 | 0 | 0 |
| 72h | 3 | 0 | 0 | 0 |
| KP1050 |  |  |  |  |
| 12h | 15 | 13 | 6 | 1 |
| 24h | 15 | 10 | 3 | 1 |
| 36h | 13 | 10 | 3 | 0 |
| 48h | 13 | 9 | 2 | 0 |
| 60h | 13 | 9 | 2 | 0 |
| 72h | 13 | 9 | 2 | 0 |
| KPNJ2 |  |  |  |  |
| 12h | 14 | 12 | 9 | 3 |
| 24h | 12 | 9 | 7 | 1 |
| 36h | 11 | 9 | 3 | 0 |
| 48h | 11 | 8 | 3 | 0 |
| 60h | 11 | 8 | 3 | 0 |
| 72h | 11 | 8 | 3 | 0 |

Table S4. The names, host species, accession numbers, carbapenemase genes and locations of IncX3 plasmids.

| Accession no. | Host species | Plasmid | Carbapenemases^a^ | Country |
| --- | --- | --- | --- | --- |
| CP027051 | *Klebsiella pneumoniae* | 20-GR-12 | - | Australia |
| CP027057 | *Klebsiella pneumoniae* | 2-GR-12 | - | Australia |
| KU963389 | *Escherichia coli* | ECO37P1 | *bla*KPC-2 | Brazil |
| KU934011 | *Serratia marcescens* | IncX-3 | *bla*KPC-3 | Italy |
| LT216437 | *Klebsiella pneumoniae* | KPN207_p1 | - | United Kingdom |
| CP034847 | *Escherichia coli* | p103-2-5 | *bla*NDM-5 | China |
| MF344558 | *Klebsiella pneumoniae* | p10677-NDM | *bla*NDM-1 | China |
| MG825384 | *Escherichia coli* | p1079-NDM | *bla*NDM-5 | China |
| KP987216 | *Citrobacter freundii* | p112298-NDM | *bla*NDM-1 | China |
| MF344560 | *Enterobacter hormaechei* | p128379 | *bla*NDM-1 | China |
| MH917281 | *Klebsiella pneumoniae* | p14504-NDM | *bla*NDM-1 | China |
| CP019073 | *Escherichia coli* | p1493 | *bla*NDM-5 | China |
| CP026727 | *Escherichia coli* | p266917 | *bla*OXA-181 | United Kingdom |
| CP019775 | *Klebsiella pneumoniae* | p3_1 | - | Switzerland |
| CP010395 | *Klebsiella pneumoniae* | p34618 | - | USA |
| KY913899 | *Klebsiella oxytoca* | p3-NDM | *bla*NDM-1 | China |
| KT362706 | *Klebsiella pneumoniae* | p45-IncX3 | *bla*KPC-3 | Italy |
| CP011983 | *Klebsiella pneumoniae* | p500_1420 | - | USA |
| KY978629 | *Cronobacter sakazakii* | p505108-NDM | *bla*NDM-1 | China |
| CP034284 | *Klebsiella pneumoniae* | p72_OXA181_X3 | *bla*OXA-181 | USA |
| MG825368 | *Escherichia coli* | p787-NDM | *bla*NDM-5 | China |
| MG825382 | *Escherichia coli* | p977-NDM | *bla*NDM-5 | China |
| MH917282 | *Klebsiella pneumoniae* | pA457-NDA | *bla*NDM-1 | China |
| MH917283 | *Klebsiella pneumoniae* | pA575-NDM | *bla*NDM-1 | China |
| KX214671 | *Escherichia coli* | pABC133-NDM | *bla*NDM-7 | United Arab Emirates |
| KX214670 | *Escherichia coli* | pABC218-NDM | *bla*NDM-7 | United Arab Emirates |
| KX833071 | *Escherichia coli* | pAD-19R | *bla*NDM-17 | China |
| CP024806 | *Escherichia coli* | pAMA1167-OXA-181 | *bla*OXA-181 | Denmark |
| MK317995 | *Klebsiella pneumoniae* | pAN65 | *bla*NDM-5 | China |
| CP025007 | *Klebsiella pneumoniae* | pAUSMDU3562 | - | Australia |
| CP022694 | *Klebsiella pneumoniae* | pAUSMDU8079 | - | Australia |
| CP022575 | *Klebsiella pneumoniae* | pBIC-1b | - | France |
| MF679143 | *Escherichia coli* | pBJ114-46 | *bla*NDM-5 | China |
| MF679147 | *Escherichia coli* | pBJ114T-190 | *bla*NDM-5 | China |
| CP020839 | *Klebsiella pneumoniae* | pBK13043 | - | USA |
| KY659387 | *Citrobacter freundii* | pCfr-30 | *bla*KPC-3 | Italy |
| KU647721 | *Escherichia coli* | pCQ02-121 | *bla*NDM-5 | China |
| KY659388 | *Citrobacter freundii* | pCr-145 | *bla*KPC-3 | Italy |
| CP024820 | *Citrobacter freundii* | pCRCB-101 | *bla*NDM-5 | South Korea |
| CP024833 | *Escherichia coli* | pCREC-532 | *bla*NDM-7 | South Korea |
| CP024828 | *Escherichia coli* | pCREC-544 | *bla*NDM-1 | South Korea |
| CP024825 | *Escherichia coli* | pCREC-591 | *bla*NDM-5 | South Korea |
| CP024818 | *Escherichia coli* | pCREC-629 | *bla*NDM-7 | South Korea |
| KX960109 | *Escherichia coli* | pCREC-A6-NDM | *bla*NDM-5 | China |
| KX960110 | *Escherichia coli* | pCREC-TJ2-NDM | *bla*NDM-5 | China |
| CP024814 | *Enterobacter sp.* | pCRENT-193 | *bla*NDM-1 | South Korea |
| **MG702491** | ***Escherichia coli*** | **pD6-OXA** | ***bla*NDM-5;**  ***bla*OXA-181** | **South Korea** |
| JN935899 | *Escherichia coli* | pEC14_35 | - | USA |
| KX618702 | *Escherichia coli* | pEC-147 | - | The Netherlands |
| KX618701 | *Escherichia coli* | pEC-170 | - | The Netherlands |
| KX618700 | *Escherichia coli* | pEC-172 | - | The Netherlands |
| KT824791 | *Escherichia coli* | pEc1929 | *bla*NDM-5 | China |
| KX618699 | *Escherichia coli* | pEC-213 | - | The Netherlands |
| MG893567 | *Escherichia coli* | pEC21-OXA-181 | *bla*OXA-181 | China |
| KX618698 | *Escherichia coli* | pEC-243 | - | The Netherlands |
| KX618704 | *Escherichia coli* | pEC-244 | - | The Netherlands |
| CP035125 | *Escherichia coli* | pEC25_NDM-7 | *bla*NDM-7 | China |
| CP020090 | *Enterobacter cloacae* | pEC27 | *bla*NDM-1 | Viet Nam |
| CP018951 | *Escherichia coli* | pEC276 | - | United Kingdom |
| KR822247 | *Escherichia coli* | pEc2A | *bla*NDM-1 | Brazil |
| KX618697 | *Escherichia coli* | pEC-393 | - | The Netherlands |
| MG545911 | *Escherichia coli* | pEC463-NDM5 | *bla*NDM-5 | China |
| KX470735 | *Escherichia coli* | pEC50-NDM7 | *bla*NDM-7 | China |
| KX470734 | *Escherichia coli* | pEC55-NDM4 | *bla*NDM-4 | China |
| KX507346 | *Escherichia coli* | pECNDM101 | *bla*NDM-5 | China |
| KX618696 | *Escherichia coli* | pEC-NRS18 | - | The Netherlands |
| CP023260 | *Escherichia coli* | pEco70745 | *bla*NDM-7 | Sweden |
| KX683284 | *Escherichia coli* | pECSEV_02 | *bla*KPC-2 | Korea |
| MH061381 | *Enterobacter cloacae* | pEn_NDM | *bla*NDM-1 | China |
| MG833403 | *Enterobacter asburiae* | pEnas-80654cz | *bla*NDM-4 | Czech Republic |
| MG833402 | *Enterobacter cloacae* | pEncl-44578cz | *bla*NDM-4 | Czech Republic |
| MG252892 | *Enterobacter cloacae* | pEncl-922cz | *bla*NDM-4 | Czech Republic |
| MG833404 | *Kluyvera intermedia* | pEnin-51781cz | *bla*NDM-4 | Czech Republic |
| MG833405 | *Escherichia coli* | pEsco-4382cz | *bla*NDM-4 | Czech Republic |
| MG252891 | *Escherichia coli* | pEsco-5256cz | *bla*NDM-5 | Czech Republic |
| CP034133 | *Klebsiella quasipneumoniae* | pG4584 | *bla*NDM-5 | USA |
| CP028182 | *Klebsiella pneumoniae* | pGMI16-005 | - | USA |
| CP031138 | *Escherichia coli* | pGMI17-003 | *bla*NDM-7 | USA |
| KY296103 | *Enterobacter cloacae* | pHN84NDM | *bla*NDM-1 | China |
| MH594478 | *Citrobacter freundii* | pIBAC | *bla*KPC-3 | Czechia |
| KY499796 | *Klebsiella pneumoniae* | pIN03-01 | - | South Korea |
| CP035910 | *Klebsiella pneumoniae* | pIncX3-1 | - | India |
| CP036195 | *Klebsiella pneumoniae* | pIncX3-2 | - | India |
| JN247852 | *Klebsiella pneumoniae* | pIncX-SHV | - | Italy |
| KM400601 | *Klebsiella pneumoniae* | pJEG027 | *bla*NDM-4 | Australia |
| MH523639 | *Escherichia coli* | pJN05NDM7 | *bla*NDM-7 | China |
| CP022351 | *Klebsiella michiganensis* | pK516_NDM5 | *bla*NDM-5 | China |
| CP023188 | *Klebsiella michiganensis* | pK518_NDM5 | *bla*NDM-5 | China |
| CP026476 | *Escherichia coli* | pKBN10P04869C | *bla*OXA-181 | South Korea |
| MG833406 | *Klebsiella oxytoca* | pKlox-45574cz | *bla*NDM-5 | Czech Republic |
| MG228426 | *Escherichia coli* | pKP_BO_OXA-181 | *bla*OXA-181 | Italy |
| KU314941 | *Klebsiella pneumoniae* | pKP04NDM | *bla*NDM-1 | China |
| KX756453 | *Klebsiella pneumoniae* | pKP1194a | *bla*KPC-2 | Brazil |
| CP003997 | *Klebsiella pneumoniae* | pKP13d | *bla*KPC-2 | Brazil |
| MF150120 | *Klebsiella pneumoniae* | pKP64477d | *bla*KPC-2 | Brazil |
| MK264770 | *Klebsiella pneumoniae* | pKP89 | *bla*KPC-2 | Brazil |
| KX348144 | *Klebsiella pneumoniae* | pKPC_Kp01 | *bla*KPC-2 | Korea |
| CP009776 | *Klebsiella pneumoniae* | pKPC-def | *bla*KPC-3 | USA |
| JX104759 | *Klebsiella pneumoniae* | pKPC-NY79 | *bla*KPC-2 | China |
| CP012990 | *Klebsiella pneumoniae* | pKpN01-NDM7 | *bla*NDM-7 | Canada |
| CP012995 | *Klebsiella pneumoniae* | pKpN06-NDM7 | *bla*NDM-7 | Canada |
| KC311431 | *Klebsiella pneumoniae* | pKPN5047 | *bla*NDM-1 | China |
| CP008799 | *Klebsiella pneumoniae* | pKPN-819 | - | USA |
| JX461340 | *Klebsiella pneumoniae* | pKpS90 | *bla*KPC-2 | France |
| CP031373 | *Klebsiella pneumoniae* | pKpvST101 | - | United Kingdom |
| KT005457 | *Klebsiella variicola* | pKS22 | *bla*OXA-181 | Switzerland |
| CP034323 | *Klebsiella pneumoniae* | pKSH203-NDM | *bla*NDM-1 | China |
| KX214669 | *Escherichia coli* | pKW53T | *bla*NDM-7 | United Arab Emirates |
| CP034748 | *Escherichia coli* | pL100 | *bla*NDM-5 | China |
| CP034591 | *Escherichia coli* | pL37 | *bla*NDM-5 | China |
| CP034730 | *Escherichia coli* | pL41 | *bla*NDM-5 | China |
| CP034737 | *Escherichia coli* | pL53 | *bla*NDM-5 | China |
| CP034744 | *Escherichia coli* | pL65 | *bla*NDM-5 | China |
| MG773377 | *Escherichia coli* | pLSB54-NDM-5 | *bla*NDM-5 | China |
| AP018141 | *Escherichia coli* | pM110_X3 | *bla*NDM-7 | Japan |
| AP018831 | *Enterobacter hormaechei* | pM206-OXA181 | *bla*OXA-181 | Japan |
| AP018142 | *Escherichia coli* | pM213_X3 | *bla*NDM-4 | Japan |
| AP018146 | *Escherichia coli* | pM216_X3 | *bla*NDM-4 | Japan |
| AP018836 | *Escherichia coli* | pM513-OXA181 | *bla*OXA-181 | Japan |
| AP018837 | *Escherichia coli* | pM517-OXA181 | *bla*OXA-181 | Japan |
| AP018838 | *Escherichia coli* | pM518-OXA181 | *bla*OXA-181 | Japan |
| AP018571 | *Citrobacter freundii* | pMH16-522D | *bla*NDM-1 | Japan |
| CP010881 | *Escherichia coli* | pMNCRE44 | *bla*KPC-3 | USA |
| CP018435 | *Klebsiella pneumoniae* | pMNCRE53 | - | USA |
| CP018423 | *Klebsiella pneumoniae* | pMNCRE69 | - | USA |
| CP018429 | *Klebsiella pneumoniae* | pMNCRE78 | - | USA |
| MH349095 | *Escherichia coli* | pMTC948 | *bla*NDM-5 | China |
| MH341575 | *Klebsiella pneumoniae* | pMYKLB95 | *bla*NDM-5 | China |
| KF220657 | *Klebsiella pneumoniae* | pNDM_MGR194 | *bla*NDM-5 | China |
| CP028786 | *Klebsiella pneumoniae* | pNDM1_020049 | *bla*NDM-1 | China |
| CP031884 | *Klebsiella pneumoniae* | pNDM1_095845 | *bla*NDM-1 | China |
| MF458176 | *Escherichia coli* | pNDM-20 | *bla*NDM-20 | China |
| CP025948 | *Escherichia coli* | pNDM21_020023 | *bla*NDM-21 | China |
| CP026577 | *Escherichia coli* | pNDM5_005237 | *bla*NDM-5 | China |
| CP028577 | *Escherichia coli* | pNDM5_005784 | *bla*NDM-5 | China |
| CP032424 | *Escherichia coli* | pNDM5_020001 | *bla*NDM-5 | China |
| CP032889 | *Escherichia coli* | pNDM5_020022 | *bla*NDM-5 | China |
| CP034957 | *Escherichia coli* | pNDM5_020026 | *bla*NDM-5 | China |
| CP033399 | *Escherichia coli* | pNDM5_020031 | *bla*NDM-5 | China |
| CP034965 | *Escherichia coli* | pNDM5_020032 | *bla*NDM-5 | China |
| CP031725 | *Enterobacter hormaechei* | pNDM5_020038 | *bla*NDM-5 | China |
| CP028536 | *Enterobacter hormaechei* | pNDM5_020042 | *bla*NDM-5 | China |
| CP028781 | *Klebsiella pneumoniae* | pNDM5_020046 | *bla*NDM-5 | China |
| CP027204 | *Escherichia coli* | pNDM5_025943 | *bla*NDM-5 | China |
| CP036179 | *Escherichia coli* | pNDM5_025970 | *bla*NDM-5 | China |
| CP036312 | *Enterobacter hormaechei* | pNDM5_090011 | *bla*NDM-5 | China |
| MH094148 | *Escherichia coli* | pNDM-5_A0917122 | *bla*NDM-5 | Korea |
| **KX674681** | ***Escherichia coli*** | **pNDM5_AS** | ***bla*NDM-5** | **China** |
| KU761328 | *Klebsiella pneumoniae* | pNDM5_IncX3 | *bla*NDM-5 | USA |
| KY435936 | *Escherichia coli* | pNDM5_WCHEC0215 | *bla*NDM-5 | China |
| CP033057 | *Morganella morganii* | pNDM5-L241 | *bla*NDM-5 | China |
| CP036205 | *Escherichia coli* | pNDM5-L725 | *bla*NDM-5 | China |
| MK308632 | *Klebsiella pneumoniae* | pNDM5-LDR | *bla*NDM-5 | USA |
| KX447767 | *Escherichia coli* | pNDM5-NJ-IncX3 | *bla*NDM-5 | USA |
| CP029386 | *Klebsiella pneumoniae* | pNDM6_040074 | *bla*NDM-6 | China |
| MF511773 | *Klebsiella pneumoniae* | pNDM-A1 | *bla*NDM-1 | South Korea |
| MF415608 | *Enterobacter cloacae* | pNDM-BJ03 | *bla*NDM-1 | China |
| MG591703 | *Escherichia coli* | pNDM-EC36 | *bla*NDM-5 | China |
| KP765744 | *Enterobacter cloacae* | pNDM-ECN49 | *bla*NDM-1 | China |
| KF976405 | *Enterobacter cloacae* | pNDM-HF727 | *bla*NDM-1 | China |
| MH234509 | *Escherichia coli* | pNDM-HK2967 | *bla*NDM-5 | China |
| MH234508 | *Klebsiella pneumoniae* | pNDM-HK2998 | *bla*NDM-5 | China |
| MH234507 | *Escherichia coli* | pNDM-HK3218 | *bla*NDM-5 | China |
| MH234506 | *Enterobacter cloacae* | pNDM-HK3473 | *bla*NDM-5 | China |
| MH234505 | *Escherichia coli* | pNDM-HK3694 | *bla*NDM-1 | China |
| MH234504 | *Klebsiella pneumoniae* | pNDM-HK3706 | *bla*NDM-5 | China |
| MH234503 | *Escherichia coli* | pNDM-HK3712 | *bla*NDM-5 | China |
| MH234502 | *Escherichia coli* | pNDM-HK3774 | *bla*NDM-5 | China |
| MH234501 | *Klebsiella pneumoniae* | pNDM-HK3816 | *bla*NDM-5 | China |
| MH234500 | *Klebsiella pneumoniae* | pNDM-HK3819 | *bla*NDM-5 | China |
| MH234499 | *Escherichia coli* | pNDM-HK3836 | *bla*NDM-5 | China |
| MH234498 | *Escherichia coli* | pNDM-HK3855 | *bla*NDM-5 | China |
| MH234497 | *Escherichia coli* | pNDM-HK3871 | *bla*NDM-5 | China |
| JX104760 | *Klebsiella pneumoniae* | pNDM-HN380 | *bla*NDM-1 | China |
| KU167608 | *Escherichia coli* | pNDM-QD28 | *bla*NDM-5 | China |
| KU167609 | *Escherichia coli* | pNDM-QD29 | *bla*NDM-5 | China |
| CP025041 | *Klebsiella pneumoniae* | pNU-CRE047 | - | USA |
| KP776609 | *Escherichia coli* | pOM26 | *bla*NDM-7 | United Arab Emirates |
| KP400525 | *Escherichia coli* | pOXA181 | *bla*OXA-181 | China |
| KX523903 | *Klebsiella pneumoniae* | pOXA-181_29144 | *bla*OXA-181 | Czech Republic |
| KX894452 | *Escherichia coli* | pOXA-181-IHIT35346 | *bla*OXA-181 | Germany |
| MF072961 | *Citrobacter freundii* | pP10159 | *bla*NDM-1 | China |
| MF547511 | *Escherichia coli* | pP744T-NDM5 | *bla*NDM-5 | China |
| MF547509 | *Escherichia coli* | pP785-NDM5 | *bla*NDM-5 | China |
| MF547507 | *Escherichia coli* | pP788A-NDM5 | *bla*NDM-5 | China |
| MF547508 | *Escherichia coli* | pP855-NDM5 | *bla*NDM-5 | China |
| **MH917280** | ***Klebsiella pneumoniae*** | **pQDE2-NDM** | ***bla*NDM-5** | **China** |
| **MK256964** | ***Escherichia coli*** | **pR15_NDM-5** | ***bla*NDM-5** | **China** |
| KF877335 | *Raoultella planticola* | pRJA274 | *bla*NDM-1 | China |
| KX023261 | *Escherichia coli* | pSCE516 | *bla*NDM-5 | China |
| MH161191 | *Klebsiella pneumoniae* | pSCKLB138 | *bla*NDM-5 | China |
| MH781720 | *Klebsiella pneumoniae* | pSCKLB684 | *bla*NDM-5 | China |
| CP028718 | *Klebsiella pneumoniae* | pSCM96-2 | *bla*NDM-19 | China |
| MH105050 | *Salmonella enterica* | pSL131_IncX3 | *bla*NDM-1 | China |
| MH105052 | *Escherichia coli* | pSL131T_IncX3 | *bla*NDM-1 | China |
| MG570092 | *Escherichia coli* | pSTIB_IncX3_OXA_181 | *bla*OXA-181 | Czech Republic |
| CP029245 | *Escherichia coli* | pTB203 | *bla*NDM-5 | China |
| MH107030 | *Klebsiella pneumoniae* | pTBCZNDM01 | *bla*NDM-5 | China |
| MH143074 | *Escherichia coli* | pTBCZNDM03 | *bla*NDM-1 | China |
| KY499797 | *Klebsiella pneumoniae* | pTH02-34 | - | South Korea |
| KY930325 | *Klebsiella pneumoniae* | pUCLAKPC2 | *bla*KPC-3 | USA |
| CP012563 | *Klebsiella pneumoniae* | pUCLAOXA232 | - | USA |
| CP011987 | *Klebsiella pneumoniae* | pUHKPC07 | - | USA |
| LC056587 | *Escherichia coli* | pV301-b | - | India |
| CP028705 | *Escherichia coli* | pVH1 | *bla*NDM-5 | China |
| JX254913 | *Citrobacter freundii* | pYE315203 | *bla*NDM-1 | China |
| KX094555 | *Escherichia coli* | pZHDC33 | *bla*NDM-13 | China |
| **KY041843** | ***Escherichia coli*** | **pZHDC40** | ***bla*NDM-5** | **China** |
| MG252893 | *Raoultella ornithinolytica* | Ror-30818cz | *bla*NDM-1 | Czech Republic |
| CP021759 | *Klebsiella pneumoniae* | tig00000002-1 | *bla*NDM-7 | USA |
| CP021715 | *Klebsiella pneumoniae* | tig00000002-2 | - | USA |
| CP020111 | *Klebsiella pneumoniae* | tig00000003-1 | - | USA |
| CP021542 | *Klebsiella pneumoniae* | tig00000003-2 | - | USA |
| CP020074 | *Klebsiella pneumoniae* | tig00000004 | - | USA |
| CP026758 | *Klebsiella aerogenes* | tig00000058 | - | USA |
| CP021534 | *Escherichia coli* | tig00000221 | *bla*NDM-7 | USA |
| CP021738 | *Escherichia coli* | tig00000260 | *bla*NDM-5 | USA |
| CP021836 | *Klebsiella pneumoniae* | tig00000583 | - | USA |
| CP021692 | *Escherichia coli* | tig00001251 | *bla*NDM-5 | USA |
| CP021682 | *Escherichia coli* | tig00003144 | *bla*NDM-7 | USA |
| LR130550 | *Klebsiella pneumoniae* | unnamed01 | - | Australia |
| CP025215 | *Klebsiella pneumoniae* | unnamed02 | *bla*NDM-7 | China |
| CP014006 | *Klebsiella pneumoniae* | unnamed03 | *bla*NDM-5 | China |
| CP032190 | *Klebsiella pneumoniae* | unnamed04 | - | USA |
| CP029735 | *Citrobacter sp.* | unnamed05 | - | USA |
| CP023897 | *Escherichia coli* | unnamed06 | *bla*OXA-181 | USA |
| CP029100 | *Klebsiella pneumoniae* | unnamed07 | - | USA |
| CP035334 | *Escherichia coli* | unnamed08 | - | Brazil |
| CP027700 | *Klebsiella pneumoniae* | unnamed09 | *bla*KPC-2 | USA |
| CP015825 | *Klebsiella pneumoniae* | unnamed10 | - | Switzerland |
| **MK715437** | ***Klebsiella pneumoniae*** | **pNDM5-SCNJ1** | ***bla*NDM-5** | **China** |

Plasmids clustered with pNDM5-SCNJ1 are shown in bold; “-” refers to the corresponding plasmid carrying no carbapenemase gene.

**References**

[1] Krishnaraju M, Kamatchi C, Jha AK, Devasena N, Vennila R, Sumathi G, et al. Complete sequencing of an IncX3 plasmid carrying *bla*_NDM-5_ allele reveals an early stage in the dissemination of the *bla*_NDM_ gene. Indian J Med Microbiol. 2015;33:30-8.

[2] Balm MN, La MV, Krishnan P, Jureen R, Lin RT, Teo JW. Emergence of *Klebsiella pneumoniae* co-producing NDM-type and OXA-181 carbapenemases. Clin Microbiol Infect. 2013;19:E421-3.

[3] Hammerum AM, Hansen F, Olesen B, Struve C, Holzknecht BJ, Andersen PS, et al. Investigation of a possible outbreak of NDM-5-producing ST16 *Klebsiella pneumoniae* among patients in Denmark with no history of recent travel using whole-genome sequencing. J Glob Antimicrob Resist. 2015;3:219-21.

[4] Bathoorn E, Rossen JW, Lokate M, Friedrich AW, Hammerum AM. Isolation of an NDM-5-producing ST16 *Klebsiella pneumoniae* from a Dutch patient without travel history abroad, August 2015. Euro Surveill. 2015;20.

[5] Cho SY, Huh HJ, Baek JY, Chung NY, Ryu JG, Ki CS, et al. *Klebsiella pneumoniae* co-producing NDM-5 and OXA-181 carbapenemases, South Korea. Emerg Infect Dis. 2015;21:1088-9.

[6] Khalifa HO, Soliman AM, Ahmed AM, Shimamoto T, Shimamoto T. NDM-4- and NDM-5-Producing *Klebsiella pneumoniae* Coinfection in a 6-Month-Old Infant. Antimicrob Agents Chemother. 2016;60:4416-7.

[7] Li A, Yang Y, Miao M, Chavda KD, Mediavilla JR, Xie X, et al. Complete Sequences of mcr-1-Harboring Plasmids from Extended-Spectrum-beta-Lactamase- and Carbapenemase-Producing Enterobacteriaceae. Antimicrob Agents Chemother. 2016;60:4351-4.

[8] Du H, Chen L, Tang Y-W, Kreiswirth BN. Emergence of the mcr-1 colistin resistance gene in carbapenem-resistant Enterobacteriaceae. The Lancet Infectious Diseases. 2016;16:287-8.

[9] Liu PP, Liu Y, Wang LH, Wei DD, Wan LG. Draft Genome Sequence of an NDM-5-Producing *Klebsiella pneumoniae* Sequence Type 14 Strain of Serotype K2. Genome Announc. 2016;4.

[10] Perez-Moreno MO, Ortega A, Perez-Vazquez M, Centelles-Serrano MJ, Bautista V, Escrig-Monfort C, et al. Simultaneous colonisation by ST340 *Klebsiella pneumoniae* producing NDM-5 and ST399 Escherichia coli producing NDM-7. Int J Antimicrob Agents. 2016;48:464-6.

[11] He T, Wang Y, Sun L, Pang M, Zhang L, Wang R. Occurrence and characterization of *bla*_NDM-5_-positive *Klebsiella pneumoniae* isolates from dairy cows in Jiangsu, China. J Antimicrob Chemother. 2017;72:90-4.

[12] Howard JC, Creighton J, Heffernan H, Werno A. Evidence of transmission of an NDM-5-producing *Klebsiella pneumoniae* in a healthcare facility in New Zealand. J Antimicrob Chemother. 2017;72:949-51.

[13] Xiao SZ, Wang S, Wu WM, Zhao SY, Gu FF, Ni YX, et al. The Resistance Phenotype and Molecular Epidemiology of *Klebsiella pneumoniae* in Bloodstream Infections in Shanghai, China, 2012-2015. Front Microbiol. 2017;8:250.

[14] Yoon EJ, Kang DY, Yang JW, Kim D, Lee H, Lee KJ, et al. New Delhi Metallo-Beta-Lactamase-Producing Enterobacteriaceae in South Korea Between 2010 and 2015. Front Microbiol. 2018;9:571.

[15] Hornsey M, Phee L, Wareham DW. A novel variant, NDM-5, of the New Delhi metallo-beta-lactamase in a multidrug-resistant *Escherichia coli* ST648 isolate recovered from a patient in the United Kingdom. Antimicrob Agents Chemother. 2011;55:5952-4.

[16] Rahman M, Shukla SK, Prasad KN, Ovejero CM, Pati BK, Tripathi A, et al. Prevalence and molecular characterisation of New Delhi metallo-beta-lactamases NDM-1, NDM-5, NDM-6 and NDM-7 in multidrug-resistant Enterobacteriaceae from India. Int J Antimicrob Agents. 2014;44:30-7.

[17] Pitart C, Sole M, Roca I, Roman A, Moreno A, Vila J, et al. Molecular characterization of *bla*_NDM-5_ carried on an IncFII plasmid in an *Escherichia coli* isolate from a nontraveler patient in Spain. Antimicrob Agents Chemother. 2015;59:659-62.

[18] Mediavilla JR, Patrawalla A, Chen L, Chavda KD, Mathema B, Vinnard C, et al. Colistin- and Carbapenem-Resistant Escherichia coli Harboring *mcr-1* and *bla*_NDM-5_, Causing a Complicated Urinary Tract Infection in a Patient from the United States. MBio. 2016;7.

[19] Baraniak A, Izdebski R, Fiett J, Gawryszewska I, Bojarska K, Herda M, et al. NDM-producing Enterobacteriaceae in Poland, 2012-14: inter-regional outbreak of *Klebsiella pneumoniae* ST11 and sporadic cases. J Antimicrob Chemother. 2016;71:85-91.

[20] Yu H, Qu F, Shan B, Huang B, Jia W, Chen C, et al. Detection of the mcr-1 Colistin Resistance Gene in Carbapenem-Resistant Enterobacteriaceae from Different Hospitals in China. Antimicrob Agents Chemother. 2016;60:5033-5.

[21] Nakano R, Nakano A, Hikosaka K, Kawakami S, Matsunaga N, Asahara M, et al. First report of metallo-beta-lactamase NDM-5-producing *Escherichia coli* in Japan. Antimicrob Agents Chemother. 2014;58:7611-2.

[22] Huang Y, Yu X, Xie M, Wang X, Liao K, Xue W, et al. Widespread Dissemination of Carbapenem-Resistant *Escherichia coli* Sequence Type 167 Strains Harboring *bla*_NDM-5_ in Clinical Settings in China. Antimicrob Agents Chemother. 2016;60:4364-8.
